# Reversal of deficits in aged skeletal muscle during disuse and recovery in response to treatment with a secrotome product derived from partially differentiated human pluripotent stem cells

**DOI:** 10.1007/s11357-021-00423-0

**Published:** 2021-08-24

**Authors:** Dennis K. Fix, Ziad S. Mahmassani, Jonathan J. Petrocelli, Naomi M.M.P. de Hart, Patrick J. Ferrara, Jessie S. Painter, Gabriel Nistor, Thomas E. Lane, Hans S. Keirstead, Micah J. Drummond

**Affiliations:** 1 Department of Physical Therapy and Athletic Training, University of Utah, 520 Wakara Way, UT 84108 Salt Lake City, USA; 2 Department of Nutrition and Integrative Physiology, University of Utah, UT Salt Lake City, USA; 3 Molecular Medicine Program, University of Utah, Salt Lake City, UT USA; 4 Department of Neurobiology and Behavior, University of California, Irvine, CA USA; 5 Immunis, Inc, Irvine, CA USA

**Keywords:** Immune cells, Fibrosis, Stem cells, Sarcopenia, Atrophy, Inflammation

## Abstract

Aged individuals are at risk to experience slow and incomplete muscle recovery following periods of disuse atrophy. While several therapies have been employed to mitigate muscle mass loss during disuse and improve recovery, few have proven effective at both. Therefore, the purpose of this study was to examine the effectiveness of a uniquely developed secretome product (STEM) on aged skeletal muscle mass and function during disuse and recovery. Aged (22 months) male C57BL/6 were divided into PBS or STEM treatment (*n* = 30). Mice within each treatment were assigned to either ambulatory control (CON; 14 days of normal cage ambulation), 14 days of hindlimb unloading (HU), or 14 days of hindlimb unloading followed by 7 days of recovery (recovery). Mice were given an intramuscular delivery into the hindlimb muscle of either PBS or STEM every other day for the duration of their respective treatment group. We found that STEM-treated mice compared to PBS had greater soleus muscle mass, fiber cross-sectional area (CSA), and grip strength during CON and recovery experimental conditions and less muscle atrophy and weakness during HU. Muscle CD68 +, CD11b + and CD163 + macrophages were more abundant in STEM-treated CON mice compared to PBS, while only CD68 + and CD11b + macrophages were more abundant during HU and recovery conditions with STEM treatment. Moreover, STEM-treated mice had lower collagen IV and higher Pax7 + cell content compared to PBS across all experimental conditions. As a follow-up to examine the cell autonomous role of STEM on muscle, C2C12 myotubes were given STEM or horse serum media to examine myotube fusion/size and effects on muscle transcriptional networks. STEM-treated C2C12 myotubes were larger and had a higher fusion index and were related to elevated expression of transcripts associated with extracellular matrix remodeling. Our results demonstrate that STEM is a unique cocktail that possesses potent immunomodulatory and cytoskeletal remodeling properties that may have translational potential to improve skeletal muscle across a variety of conditions that adversely effect aging muscle.

**Supplementary Information:**

The online version contains supplementary material available at 10.1007/s11357-021-00423-0.

## Introduction

Periods of muscle disuse often occur in aging as a consequence of illness, injury or recovery from surgery [1–3]. These disuse events often lead to the rapid atrophy of skeletal muscle mass and weakness which is associated with poor prognosis and increased risk of re-hospitalization or further injury [4]. Age-related loss of muscle mass and strength [5] result in decreased mobility and quality of life while also increasing the risk of other co-morbidities and mortality [6–8]. Furthermore, recovery from disuse atrophy is often compromised in aged muscle, delayed or never fully achieving the previous baseline muscle size and functional quality [9, 10]. Therefore, novel therapies are needed to to mitigate muscle atrophy while improving muscle recovery following disuse especially with application to aging.

A mechanism linked to muscle dysfunction during aging is the accumulation of muscle collagen (fibrosis) in skeletal muscle [11, 12]. Excessive muscle collagen deposition impairs the functional capacity to generate force [13–16]. Recent evidence has demonstrated that the abundance of collagen in aged mouse muscle is elevated compared to their younger counterparts [17]. Muscle disuse in rodents further contributes to the accumulation of collagen [18, 19], thus raising concern that excessive fibrosis may participate in the less than optimal recovery in aging muscle. Concomitant with age-related muscle fibrosis is a decline in the muscle satellite cell pool which is critical to the ability of muscle to recover from injury [20, 21]. Moreover, aging is associated with a decline in the number and activity of myogenic satellite cells during injury which are necessary for continued myogenesis [21–23]. Therefore, it is reasonable to consider that altered satellite cell function and collagen deposition likely partly contribute to poor quality of aging muscle during disuse and recovery.

While many studies have examined mechanisms of muscle fibrosis and satellite cell function in aging there are still significant gaps in the understanding of what drives aging muscle toward this phenotype and worsened during disuse atrophy and recovery conditions. One possibility is a dysfunctional immune system associated with aging [24, 25]. Regrowth of skeletal muscle following injury or disuse atrophy require carefully timed and coordinated events involving immune cells (e.g., neutrophils, macrophages) that promote a tightly controlled pro and anti-inflammatory local environment to ensure activation and proliferation of satellite cells and proper collagen remodeling [26]. Our laboratory and others have highlighted that aged skeletal muscle is present with an impaired pro-inflammatory macrophage response following injury [27–30] or disuse [31, 32]. Interruption in the infiltration or inflammatory status of macrophage severely compromises muscle recovery and myogenic cell function and is often characterized by smaller fibers and increased collagen deposition/fibrosis [33–37]. Therefore, infiltrating muscle macrophages are critical to promote satellite cell function and optimize collagen deposition and proper cellular remodeling thereby ensuring optimal resolution of skeletal muscle.

Unfortunately, there are no known treatments that prevent atrophy during disuse and amplify muscle recovery following disuse in aged muscle [38–41]. In the current study, we tested a novel secretome-based treatment (termed STEM) to improve aged muscle during disuse atrophy and recovery. STEM is composed of mixture of molecules important to recruit immune cells while also delivering growth factors and mediators of collagen turnover necessary for satellite cell function and optimal deposition of collagen. Therefore, we suspected that this multi-component cocktail would have the capacity to specifically target age-related muscle deficits (macrophages, satellite cells), thereby promote aged skeletal muscle mass and function during disuse and recovery. We hypothesized that STEM would mitigate skeletal muscle atrophy during disuse and improve regrowth during recovery. Moreover, we also hypothesized that STEM would enhance muscle macrophage and satellite cell content while decrease muscle fibrosis.

## Materials and methods

### Animals

**Fig. 1:**
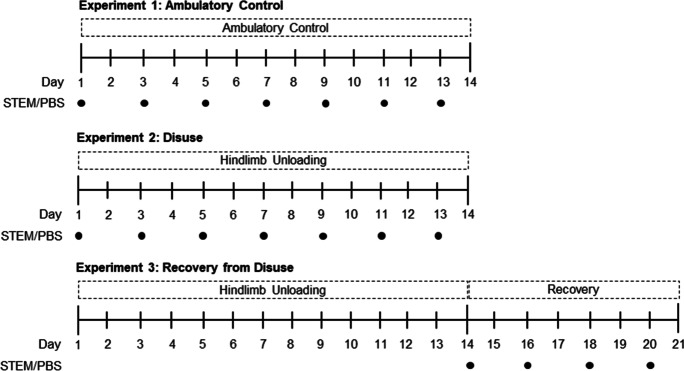
Experimental design: figure depict experimental design for all three experiments. First panel describes the design for ambulatory control conditions. Second panel describes the experimental design for hindlimb unloading conditions. Third panel describes the design for recovery following disuse atrophy conditions. Black circles indicate when PBS or STEM was injected into the right triceps surae complex

Thirty aged male C57BL/6 at 22 months of age, generously provided by National Institute on Aging mouse colony, were divided into STEM or PBS treatment groups (*n* = 15/group). Mice within each treatment were then assigned to one of 3 experimental groups: (a) ambulatory control (14 days of normal cage ambulation) (CON; *n* = 5), (b) 14 days of hindlimb unloading (HU; *n* = 5), or 14 days of hindlimb unloading followed by 7 days of recovery (Recovery; *n* = 5) (Fig. 1). All experiments were run over the course of two months in a sequential fashion. Mouse studies in experiments 1 and 2 were conducted during the course of 1 month and aged-matched mice were used for experiment 3 approximately 1 month later. Animals were housed with ad libitum access to food and water, and maintained on a 12-h light/dark cycle. All experimental procedures were conducted in accordance with the guidelines set by the Institutional Animal Care and Use Committee (IACUC) at the University of Utah.

### STEM characterization, administration, and experimental design

STEM is non-cell-based secretome derived from partially differentiated human embryonic stem cells composed of a multitude of pro-regenerative factors involved in immunomodulation, cytoskeleton remodeling, and growth factor–mediated cellular signaling (Suppl Fig. 1). It is a sterile and nonpyrogenic aqueous solution of USP grade proteins, amino acids, vitamins, and minerals containing the factors secreted by partial differentiated pluripotent stem cells. The solution does not contain live cells or any cell fragments. The factors within the secretome were determined in three independent samples by a third party (RayBiotech) using a Quantibody Multiplex ELISA platform (Human Cytokine Array Q440). The measured quantities of the targets in each group were converted in picomoles (pmol/mL) and averaged for each group and loosely categorized using a combination of Reactome, Gene Cards, and PubMed index. Many of these factors have known roles to modulate an immune response (OPN, MIF, CXCL16, GRO⍺, MCP1/CCL2, ADAM8) [42–51] and promote muscle growth either targeting the satellite cell or other supporting cells such as follistatin, insulin, IGF-binding protein 2, and IGF-binding protein-6, and TGFβ1 [52–56], while many of the other factors are important for extracellular matrix remodeling (e.g., Integrin signaling, Cathepsin, TIMP, MMP).

Mice were given a 100-μl intramuscular injection of either PBS or STEM into the right triceps surae muscle group every other day for each treatment group using a similar injection strategy as reported by Dumont and Frenette [57]. Ambulatory control and hindlimb unloaded mice received a total of six injections over the respective 14-day period treatment group duration (Fig. 1). To effectively examine STEM’s effect on muscle recovery/regrowth, a total of 4 injections were given to the animals that underwent hindlimb unloading and recovery yet only delivered during the 7-day recovery phase of the treatment (Fig. 1). All mice received the final treatment injection the day prior to being euthanized to avoid any acute treatment effects. The soleus muscle was selected for the analysis due to its higher content of resident immune cells and its sensitivity to disuse atrophy. The injection method also appears to have the most consistent effects on the soleus muscle (Suppl Fig. 2).

### Hindlimb unloading and recovery

For the hindlimb unloading and reload groups, animals underwent hindlimb unloading via tail suspension (2 animals/cage) using a modified unloading method based on the traditional Morey-Holton design for studying disuse atrophy in rodents, with some additional modifications [58]. Body weight was monitored every other day to ensure that mice did not experience excessive weight loss due to malnutrition or dehydration. Following day 14 of hindlimb unloading, animals were fasted for 5 h and then euthanized for tissue analysis. In a separate group of mice, animals underwent 14 days of hindlimb unloading and afterwards were removed from the suspension apparatus then housed in individual cages for 7 days of ambulatory recovery. Cage ambulatory controls were followed for 14 days and were housed in groups of 2–3 mice/cage. Upon completion of each treatment group, the triceps surae muscle group (soleus, plantaris, gastrocnemius) from the left and right hindlimbs were carefully dissected, weighed, prepped for immunohistochemistry, and snap frozen in liquid nitrogen cooled isopentane.

### Grip strength

To assess whole body strength, mice underwent grip strength analysis on a rodent grip strength meter (Columbus Instruments, Columbus OH). Mice were acclimated to the procedure over a course of 3 days 1 week prior to the experiments. During this acclimation period, mice were allowed to stand on the force transducer grid for a duration of 3 min. Recorded grip strength occurred on day 3. For the grip strength protocol, mice grasped the force transducer grid with their forelimbs and hindlimbs and were gently pulled by the tail across the grid by the same investigator. Five repetitions with a 5-s rest period were averaged to determine each animal’s grip strength. In experiment 1, the ambulatory control mice (CON), grip strength was determined twice: the day prior to initiating PBS or STEM treatment and on day 13 one day prior to tissue harvest. In experiment 2, hindlimb unloading (HU) mice were tested three times: the day prior to treatment, at day 7 and again on day 13 one day prior to tissue harvest. In experiment 3, recovery mice were also tested three times: the day prior to hindlimb unloading, at the end of hindlimb unloading, and on day 6 of the recovery period. All measurements were taken at 0900 and immediately prior to the treatment injection to avoid confounding effects of the injection.

### Immunofluorescence

The treated (right) soleus muscle was prepped for histology by embedding in OCT and freezing in liquid nitrogen cooled isopentane. Several cryosections were cut at a thickness of 10 µm and used for analysis of myofiber cross-sectional area (CSA) which was determined using laminin (1:100; Santa Cruz Biotechnology, Dallas, TX). Myofiber CSA was measured using semiautomatic muscle analysis with segmentation of histology, a MATLAB application (SMASH) alongside ImageJ software [32, 59]. For macrophage abundance, fiber borders were assessed either using laminin or dystrophin (1:100; Santa Cruz Biotechnology, Dallas, TX), macrophages were detected using anti-rat CD68 (1:100 Bio-Rad, Hercules, CA), anti-mouse CD11b (1:50 Biolegend, San Diego, CA) and anti-rabbit CD163 (1:100, Bio-Rad, Hercules, CA). Anti-rat secondary antibody (1:250, AF555, Invitrogen), anti-mouse secondary antibody (1: 500, A488, Invitrogen), and anti-rabbit secondary antibody (1:500, AF647, Invitrogen) were applied and then mounted in DAPI-containing mounting medium (Vector). Muscle satellite cells were stained using Pax7 (1:1000, Cell Signaling, Danvers, MA). To assess fibrosis, cryosections were stained using biotin collagen hybridizing peptide (1:100, BCHP—marker for collagen breakdown; 3Helix, Salt Lake City, UT) and Collagen IV (1:100, COLIV—marker for collagen synthesis, Abcam, Cambridge, MA). Images were analyzed using Nikon NES elements software; the intensity was set based upon PBS and then STEM images were analyzed using that intensity setting with the region of interest depicting the % of the section that stained positive for BCHP and COLIV. The ratio of these two stains is an indicator of collagen IV turnover of the muscle. All stained slides were observed with a fully automated widefield light microscope (Nikon, Tokyo, Japan) with the × 10 or × 20 objective lens. Images were taken using a high sensitivity Clara CCD camera (Belfast, UK).

### C2C12 myogenic index, myofiber size, and RNA sequencing

C2C12 myoblasts were plated in 6-well plates and upon achieving ~ 95% confluency, differentiation media DMEM supplemented with 2% horse serum and penicillin/streptomycin (ATCC, Manassas, VA) was used for 5 days to differentiate myoblasts to myotubes. Upon completion of 5 days, differentiation media was removed and replaced with media that contained varying percentages of STEM (2, 4, 8, 12, and 24%) instead of horse serum for 24 h. Control wells contained either normal differentiation media containing 2% horse serum or 4% horse serum. A serum-free control was also utilized. Following 24 h, cells were fixed and stained using MF 20 (Myosin Heavy Chain Sarcomere, DSHB, Iowa City, Iowa) and DAPI. Cells were then imaged at the University of Utah Imaging Core facility on a Nikon Eclipse Ti widefield scanning microscope. A 10 × 10 field image was obtained for each well and analyzed using ImageJ (NIH, Bethesda, MD). Myonuclear fusion was defined as nuclei in myotube/total nuclei in image field. Myotube percent area was defined as percent area of image covered by myotubes. A total of 6 replicates were used for each treatment conditions.

Based on the preliminary analysis of the most robust STEM effect on myotubes, we utilized 4% STEM and 4% horse serum (control) for bulk RNA sequencing. Cells were collected for total RNA using 1 ml of Qiazol per well and purified (using miRNAeasy Mini Kit). RNA was then treated with TURBO DNase (ThermoFisher) and purified using RNA Clean and Concentrator 5 Columns (Zymo Research). Libraries were prepared with Illumina TruSeq Stranded Total RNA Library Prep Ribo-Zero Gold (Genome Builds mm10, M_musculus_Dec_2011, GRCm38) and RNA was sequenced using Illumina NovaSeq Reagent Kit v1.5 150 × 150 bp Sequencing (100 M read-pairs). Data can be found on the Gene Expression Omnibus (GSE165110).

### Statistical and bioinformatic analysis

Results are reported as the means ± standard error. A *T*-test, one-way ANOVA, or two-way ANOVA with repeated measures was employed when appropriate. Post hoc analyses were performed with Tukey or Student–Newman–Keuls methods when appropriate. All data was assessed for normal distribution (Shapiro–Wilk test). The accepted level of significance was set at *p* < 0.05 for all analysis. Statistical analysis was performed using Prism GraphPad 7 (GraphPad Software Inc., La Jolla, CA). For RNA sequencing, differentially expressed genes were identified using a 5% false discovery rate with DESeq2 version 1.26.00. The volcano plot was generated by taking the − Log10(adj. *P*-value) on the *Y*-axis and plotting vs. the log2-fold change on the *X*-axis. The top 20 significantly decreased and top 20 increased genes were identified by taking all significantly altered transcripts (adj. *P*-value ≤ 0.05) and then sorting by log2-fold change. Values were converted out of log2 for presentation in the table.

## Results

### Effect of STEM on soleus muscle and strength in ambulatory control mice

**Fig. 2:**
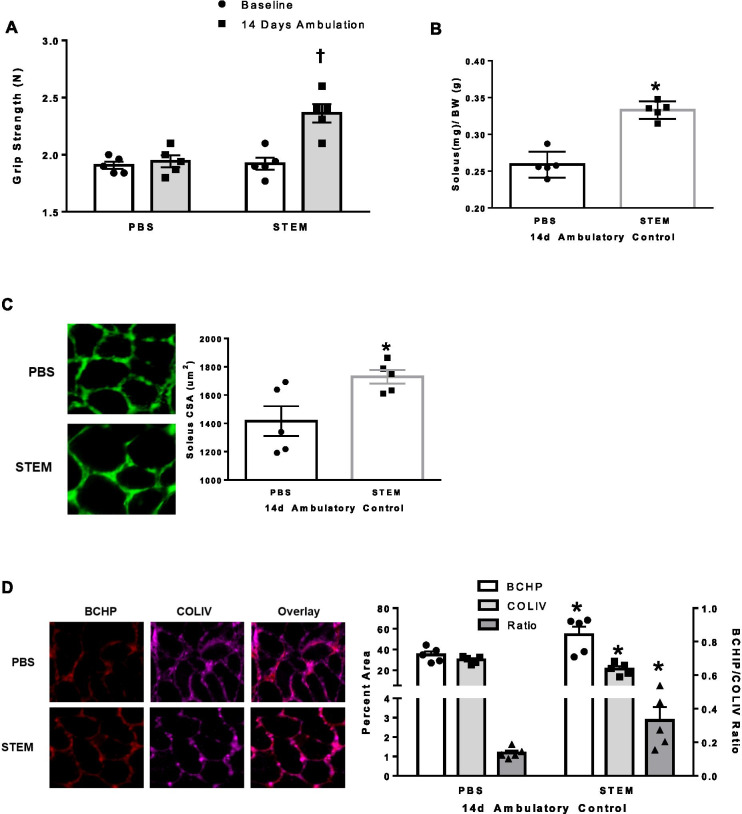
STEM effects on grip strength, mass, and collagen content during 14 days of normal cage ambulation: panels represent **A** grip strength of PBS and STEM-treated mice at baseline (1 day prior to treatment) and repeated at day 13 of normal cage ambulation (1 day prior to tissue harvest). **B** Soleus muscle mass (mg/g); **C** soleus muscle cross-sectional area (CSA) displayed in um^2^; and **D** soleus muscle BCHP, COLIV, and BCHP/COLIV ratio in PBS- and STEM-treated mice after 14 days of normal cage ambulation. Representative immunohistochemistry images are depicted next to panel. *N* = 5 mice in each group. Results are mean with standard error of the mean. Two-way ANOVA-repeated measures used for panel **A**. *T*-test between PBS and STEM (**B**–**D**). † = different from all groups. * = different to PBS

Our first goal was to determine the effect of STEM on hindlimb muscle mass and strength during 14 days of normal cage ambulation in old mice. We chose to focus our examination on the soleus due to its sensitivity to muscle atrophy during disuse and, secondly, during the course of our experiments it was the muscle most influenced by STEM (e.g., plantaris size was higher with STEM while gastrocnemius was not affected; Supplemental Fig. 2). Strikingly, STEM delivery to a single limb over the course of 14 days of normal cage ambulation resulted in higher whole body grip strength (*p* = 0.008) above baseline levels (Fig. 2A). Moreover, STEM-treated mice had greater soleus muscle mass (*p* = 0.0001) than PBS-treated mice (Fig. 2B). Consistent with enhanced soleus muscle mass, STEM-treated mice also had larger (*p* = 0.02) fiber cross-sectional area (CSA) compared to PBS-treated mice (Fig. 2C). Moreover, STEM-treated mice (compared to PBS) had elevated BCHP and lower COLIV content resulting in a higher BCHP/COLIV ratio (*p* = 0.004) (Fig. 2D) suggesting a decrease in overall soleus muscle COLIV content.

**Fig. 3:**
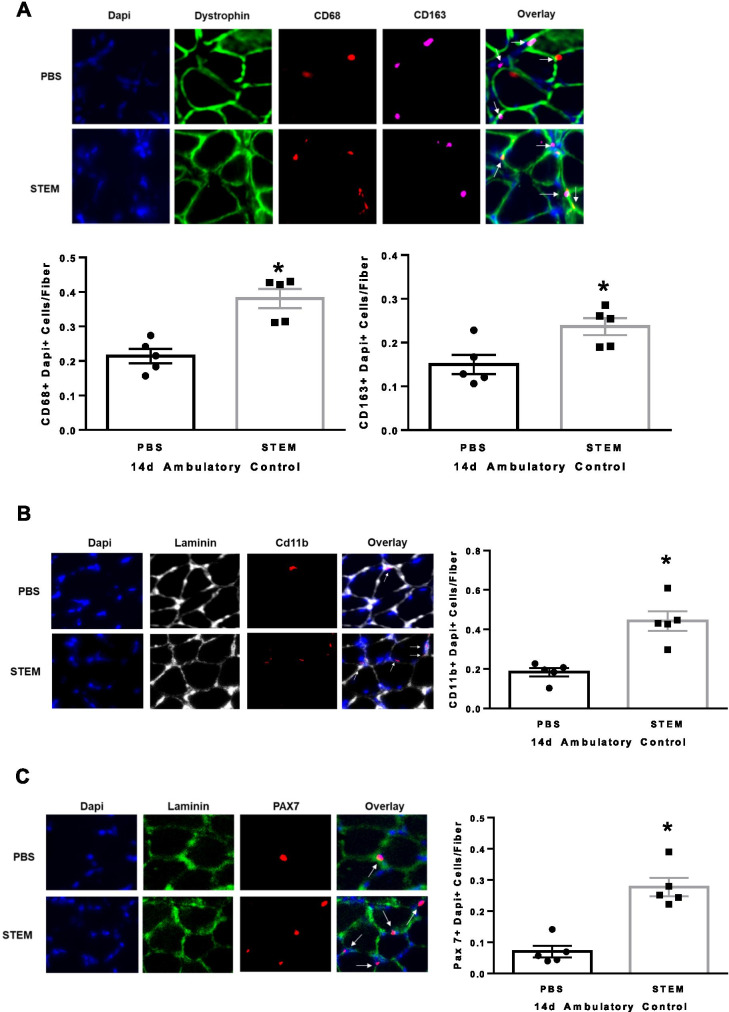
STEM effects on soleus muscle macrophage and Pax7 cell content during 14 days of normal cage ambulation:** A** CD68 + and CD163 + , **B** CD11b + , and **C** PAX7 + abundance in soleus muscle of PBS- and STEM-treated mice after 14 days of normal cage ambulation. Representative immunohistochemistry images are depicted next to panel. White arrows depict positive stained cells. Laminin in Cd11b was switched to white in ImageJ to more clearly depict the overlay. *N* = 5 mice in each group. Results are mean with standard error of the mean. *T*-test between PBS and STEM. * = different to PBS

Given the STEM cocktails unique immunomodulatory composition, we next determined if the treatment influenced macrophage content in soleus muscle. We found that STEM-treated mice had higher (*p* = 0.0002) CD68 + /DAPI + cells and CD163 + /DAPI + (*p* = 0.0002) content (Fig. 3A) compared to PBS. STEM-treated mice also had higher CD11b + abundance (*p* = 0.001) compared to PBS (Fig. 3B). Interestingly, STEM-treated mice had nearly triple the number of Pax7 + cells (*p* = 0.0001) compared to PBS-treated ambulatory control mice (Fig. 3C). There were no differences in body weight between STEM- and PBS-treated mice during the 14-day treatment period (Suppl Fig. 2D). Together, these results suggest that STEM-treated mice had higher soleus muscle mass, strength, macrophage content, Pax7 + cell abundance, and lower muscle fibrosis during 14 days of cage ambulation in aged mice.

### Effect of STEM on soleus muscle and strength after hindlimb unloading

**Fig. 4:**
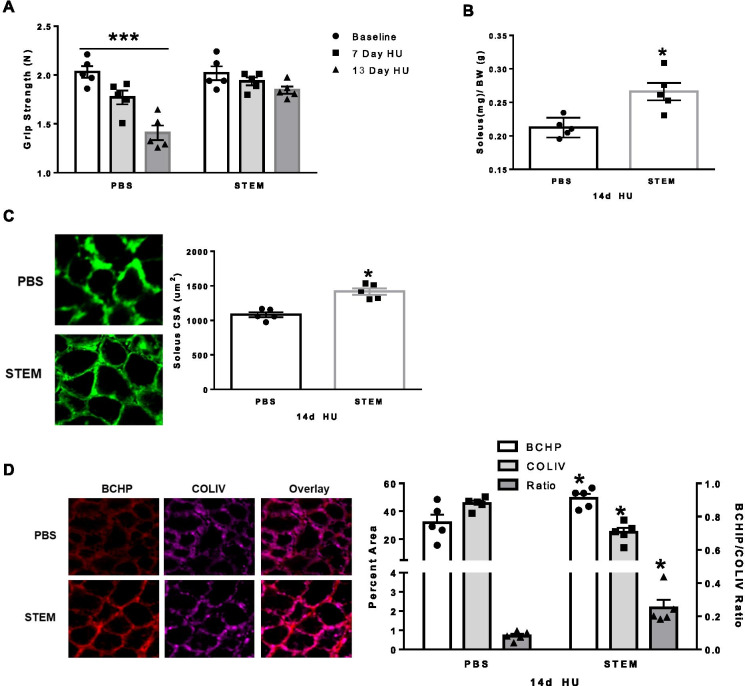
STEM effects on grip strength, muscle mass, and collagen content after hindlimb unloading: panels represent **A** grip strength of PBS- and STEM-treated mice at baseline (1 day prior to treatment), repeated at day 7 of HU (hindlimb unloading), and day 13 of HU (1 day prior to tissue harvest). **B** Soleus muscle mass (mg/g); **C** soleus muscle cross-sectional area (CSA) displayed in um^2^; and **D** soleus muscle BCHP, COLIV, and BCHP/COLIV ratio in PBS- and STEM-treated mice after 14 days of HU. Representative immunohistochemistry images are depicted next to panel. *N* = 5 mice in each group. Results are mean with standard error of the mean. Two-way ANOVA-repeated measures used for panel **A**. *T*-test between PBS and STEM (**B**–**D**). *** = all PBS groups different to each other. * = different to PBS

For the second experiment, we determined if STEM administration would mitigate muscle atrophy and weakness that is modeled as a result of 14 days of hindlimb unloading. Following hindlimb unloading, we found that STEM treatment prevented the loss of grip strength at 7- and 13-days HU when compared to HU mice treated with PBS (*p* = 0.004) (Fig. 4A). Similarly, STEM-treated mice had greater soleus size after HU (*p* = 0.006) (Fig. 4B) compared to PBS. In agreement with the muscle mass data, STEM-treated mice had larger soleus fiber CSA (*p* = 0.002) compared to PBS-treated mice (Fig. 4C). STEM-treated mice also had higher BCHP (*p* = 0.01) and lower COLIV (*p* = 0.0001) content compared to PBS-treated mice corresponding to a higher ratio of BCHP to COLIV (*p* = 0.04) in soleus muscle of HU STEM-treated mice (Fig. 4D). These results demonstrate that STEM was capable of mitigating disuse-induced atrophy and improving collagen IV turnover in aged soleus muscle when compared to PBS treatment.

**Fig. 5:**
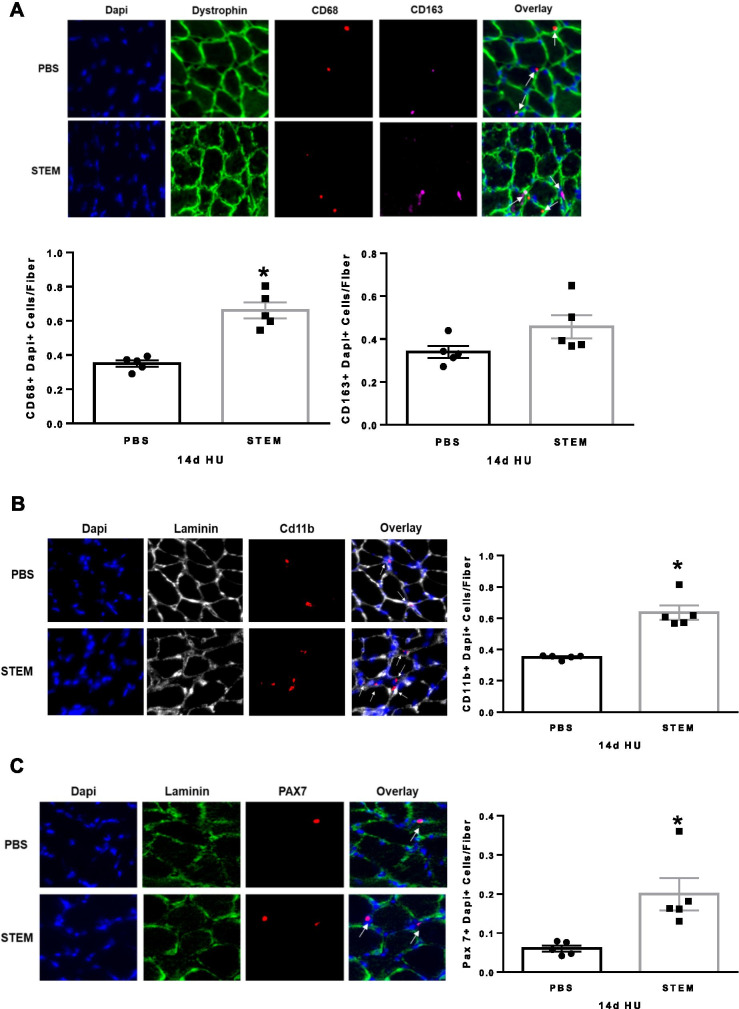
STEM effects on soleus muscle macrophage and Pax7 cell content after 14 days of hindlimb unloading:** A** CD68 + and CD163 + , **B** CD11b + , and **C** Pax7 + abundance in soleus muscle of PBS- and STEM-treated mice after 14 days of HU. Representative immunohistochemistry images are depicted next to panel. White arrows depict positive stained cells. Laminin in Cd11b was switched to white in ImageJ to more clearly depict the overlay. *N* = 5 mice in each group. Results are mean with standard error of the mean. *T*-test between PBS and STEM. * = different to PBS

We next determined if STEM modulated macrophage and Pax7 cell content in hindlimb unloaded muscle. We report that STEM-treated mice had higher abundance of macrophages as noted by CD68 + /DAPI + (*p* = 0.0009) and CD11b + /DAPI + (*p* = 0.003) cell content (Fig. 5A and B). CD163 + /DAPI + cell content was not different in the soleus muscle between STEM and PBS-treated HU mice (Fig. 5A). Lastly, STEM-treated mice had significantly higher Pax7 + cell abundance in the soleus muscle (*p* = 0.0004) compared to PBS-treated mice during HU (Fig. 5C). There were no differences in body weight between STEM- and PBS-treated mice during the 14-day HU period (Suppl Fig. 2E). Together, these results demonstrate that STEM-treated mice had higher CD11b + and CD68 + macrophages and satellite cell (Pax7 +) abundance in aged soleus muscle during disuse atrophy compared to PBS-treated mice.

### Effect of STEM on soleus muscle and strength recovery following hindlimb unloading

**Fig. 6:**
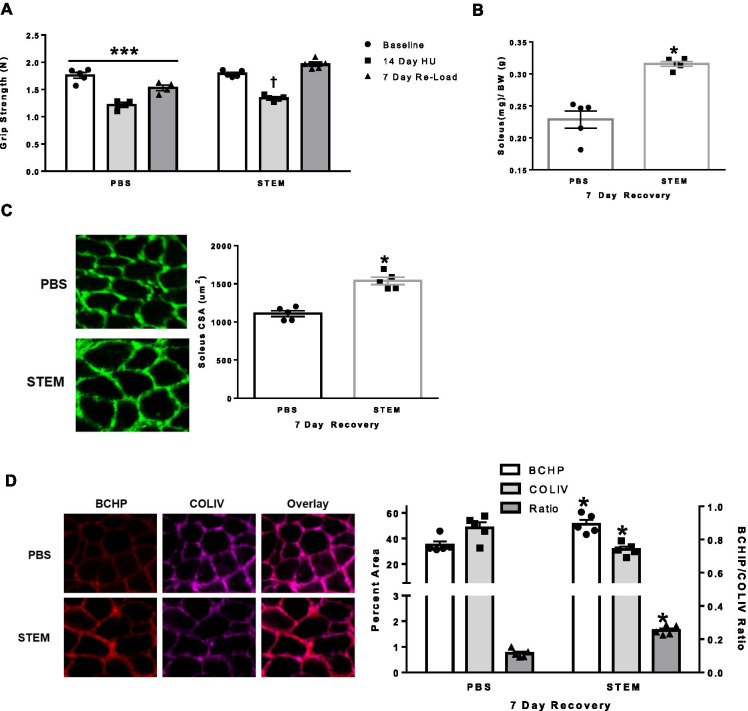
STEM effects on skeletal muscle after 7 days of recovery following hindlimb unloading: panels represent **A** grip strength of PBS- and STEM-treated mice at baseline (1 day prior to treatment), repeated at day 14 of HU (hindlimb unloading), and day 6 of RL (reload,1 day prior to tissue harvest). **B** Soleus muscle mass (mg/g); **C** soleus muscle cross-sectional area (CSA) displayed in um^2^; and **D** soleus muscle BCHP, COLIV, and BCHP/COLIV ratio in STEM- and PBS-treated mice after 7 days of recovery following 14 days of hindlimb unloading. Representative immunohistochemistry images are depicted next to panel. *N* = 5 mice in each group. Results are mean with standard error of the mean. Two-way ANOVA-repeated measures used for panel *A*. *T*-test between PBS and STEM (**B**–**D**). *** = all PBS groups different to each other. † = different to all groups. * = different to PBS

In the last experiment, we were interested if STEM would result in a greater recovery of muscle and strength following 14 days of HU compared to PBS treatment. We found that three intramuscular injections of STEM over the course of 7 days of recovery resulted in higher grip strength (*p* = 0.0001) acompared to the PBS treatment group (Fig. 6A). Moreover, STEM-treated mice had larger soleus muscle mass (*p* = 0.001) compared to PBS-treated mice (Fig. 6B). Similar to the muscle mass data, STEM-treated mice had greater soleus fiber CSA (*p* = 0.003) compared to PBS-treated mice (Fig. 6C) and was similar to the fiber CSA of PBS-treated ambulatory control mice from the first experiment. STEM-treated mice also had higher BCHP (*p* = 0.001) and lower COLIV (*p* = 0.002) content resulting in a higher ratio (*p* = 0.0009) of BCHP/COLIV (Fig. 6D). Together, these results demonstrate that STEM improved aged soleus muscle mass and promoted the growth of myofibers during recovery following disuse atrophy. Additionally, STEM-treated mice had higher collagen IV turnover and an overall net reduction in collagen IV content.

**Fig. 7:**
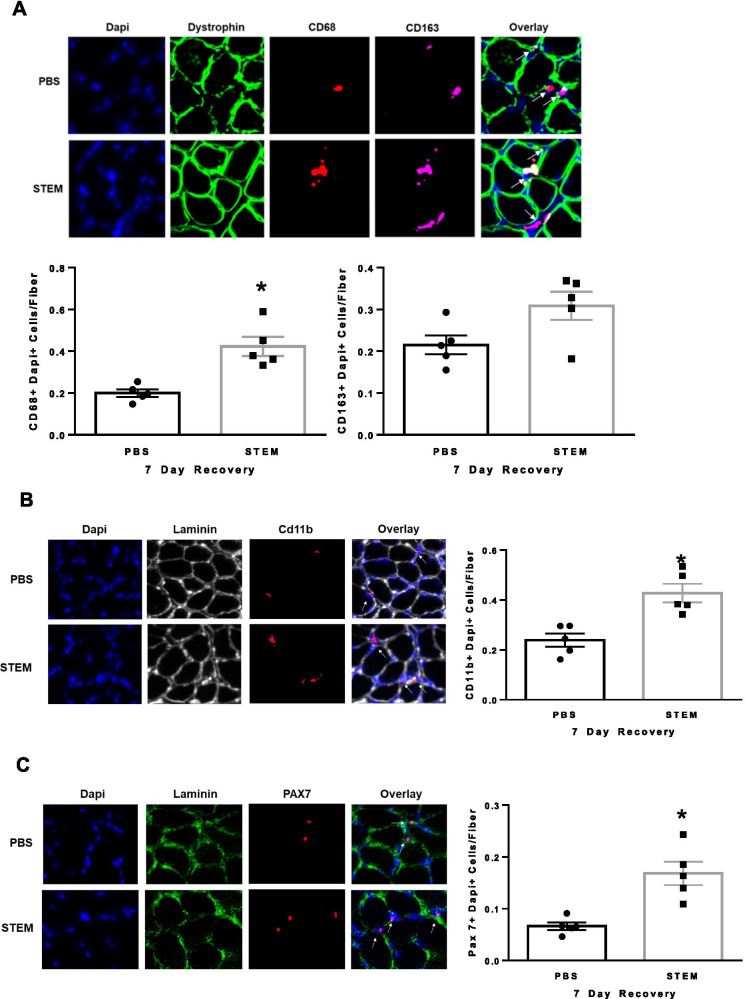
STEM effects on soleus muscle macrophage and Pax7 cell content after 7 days of recovery following hindlimb unloading:** A** CD68 + and CD163 + , **B** CD11b + , and **C** Pax7 + abundance in soleus muscle of PBS- and STEM-treated mice after 7 days of recovery following 14 days hindlimb unloading. Representative immunohistochemistry images are depicted next to panel. White arrows depict positive stained cells. Laminin in Cd11b was switched to white in ImageJ to more clearly depict the overlay. *N* = 5 mice in each group. Results are mean with standard error of the mean. *T*-test between PBS and STEM. * = different to PBS

Finally, we examined the effect of STEM on muscle macrophages and satellite cell abundance during recovery following disuse. STEM-treated mice had higher CD68 + /DAPI + (*p* = 0.0056) and CD11b + /DAPI + cells macrophage content in the soleus muscle (Fig. 7A and B). CD163 + /DAPI + cell macrophage content was not different between STEM- and PBS-treated mice during 7 days of recovery (Fig. 7A). STEM-treated mice also had an abundantly more Pax7 + cell (*p* = 0.0005) content in the soleus muscle of mice during 7 days of recovery compared to PBS-treated mice (Fig. 7C). There were no differences in body weight between STEM- and PBS-treated mice during recovery following 14d HU (Suppl Fig. 2F).Together, these results demonstrate that STEM-treated mice had higher macrophage and satellite cell abundance in aged skeletal muscle during recovery.

### Effect of STEM on C2C12 myotube fusion and size

**Fig. 8:**
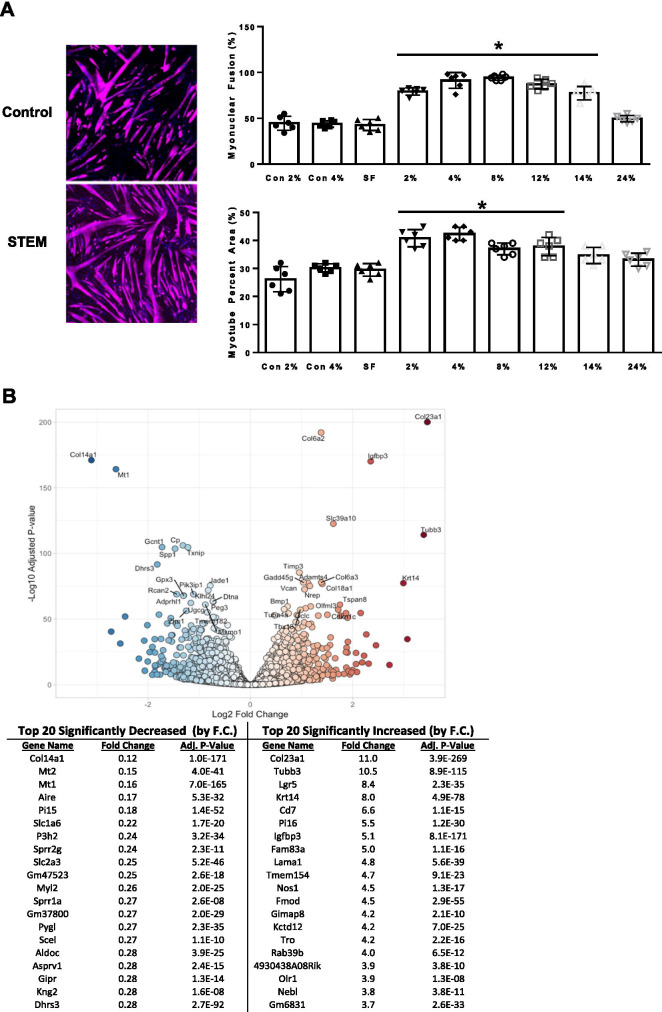
STEM effects on C2C12 myotube fusion, size, and myotube transcriptional signature: panels represent **A** quantification of controls (2% and 4% horse serum) and 2–24% STEM-treated C2C12 myotubes for myonuclear fusion (%) and myotube size. Representative images for horse serum and STEM for myonuclear fusion and myotube percent area. **B** Volcano plot and table of top significantly increased and decreased genes in STEM-treated myotubes compared to horse serum (control). SF denotes serum free media. Results are mean with standard error of the mean, one-way ANOVA. * = different from groups not under solid line

To further evaluate the effect of STEM independently on skeletal muscle, we exposed C2C12 myotubes to varying concentrations of STEM after 5 days of differentiation. As a result, we observed a robust induction of myonuclear fusion (percentage of nuclei per plate in a myotube; *p* = 0.0001) and myotube percent area (size; *p* = 0.0002) at 2, 4, 8, and 12% STEM when compared to controls (2 and 4% horse serum as well as serum-free media) (Fig. 8A). In lieu of these results, we repeated the experiments in a separate cohort of C2C12 myotubes in the presence of 4% STEM (compared to 4% horse serum) and performed RNA sequencing in order to provide insight how STEM may affect C2C12 myotubes at the transcriptional level. We report that STEM induced a transcriptional program related to cell growth and remodeling of collagen (Fig. 8B). Notable genes that were regulated pertained to collagen synthesis, degradation, and growth factors (IGF-binding protein 3, Col23a1, Col14a1, Mt2, Mt1, and Fmod). Overall, these results suggest that STEM improved myogenesis in C2C12 myotubes through the regulation of genes associated with growth factors and promotion of collagen remodeling.

## Discussion

Here, we report the unique observation that STEM treatment attenuated the loss of soleus muscle mass and fiber cross-sectional area during disuse and resulted in higher muscle mass and cross-sectional after 7 days of recovery in ambulatory control–aged mice. Moreover, these findings were supported by improved grip strength and muscle collagen IV turnover. We also report that STEM treatment was capable of modulating the content of muscle macrophages and satellite cells across all treatment groups. Together, these results suggest that STEM is a novel therapeutic that can enhance muscle size and strength and reduce collagen IV accumulation during disuse and recovery in aged mice.

Skeletal muscle is a highly adaptive tissue capable of modulating its size and functional capacity in response to changes in loading stimuli [60]. Hindlimb unloading in mice induces robust atrophy and weakness and is frequently used to model disuse atrophy and impaired muscle recovery in aging [1, 3, 10, 61]. Limited muscle size and functional reserve can lead to disability and lower quality of life in older adults [62], thereby emphasizing the need to develop therapeutics to counter these deficits in muscle structure and function. Our primary findings demonstrate that STEM administration was capable of inducing soleus muscle mass and cross-sectional area in a variety of scenarios with application to sarcopenia, muscle disuse, and age-related impairments following recovery from disuse. Interestingly, whole body grip strength was improved across all treatment groups following a local muscle administration of STEM to a single limb, suggesting that a systemic effect of STEM may have occurred (e.g., diffusion into microcirculation, release of myokines), though this will require future examinations. Furthermore, in vitro analysis demonstrated that STEM can influence muscle directly by inducing myotube hypertrophy and fusion. Therefore, we speculate that STEM promoted muscle hypertrophy directly in vitro and in vivo likely driven by the multitude of growth factors within the secretome such as follistatin, insulin, IGF-binding protein 2, and IGF-binding protein 6 [52–56].

Another major finding was that STEM-treated mice had lower muscle collagen IV content and higher CD68 + and CD11b + macrophage abundance following all three experimental conditions when compared to PBS-treated mice. A common ailment of aged skeletal muscle is elevated collagen deposition which is believed to partly contribute to impaired function during aging and poor regrowth following disuse atrophy [11, 12, 63, 64]. Moreover, dysfunctional inflammatory macrophages and an accumulation of anti-inflammatory-like (CD163 +) macrophages may underscore the poor remodeling of aged skeletal muscle [21, 23–25, 32]. Pro- and anti-inflammatory-like macrophages secrete a mileu of cytokines and growth factors that regulate satellite cell function (proliferation [65–67], differentiation [65, 66]), and collagen turnover [67]. In the current study, we demonstrated that STEM-treated mice were characterized with a heightened level of inflammatory macrophages across all experimental treatment groups. This is in line with the success of immunotherapies that promote macrophage infiltration in healthy and aged skeletal muscle and thus are effective to enhance muscle regrowth from disuse and injury [68–70]. While the only immune cells we examined were macrophages, we recognize that the STEM cocktail may be indirectly influencing muscle by regulating a multitude of immune cells important for muscle regrowth such as neutrophils and T cells. Together, these findings suggest that STEM administration at baseline and during disuse and recovery periods promoted an accumulation of CD68 + and CD11b + macrophages coinciding with lower collagen IV content which cumulatively might be related to greater muscle size expansion and heightened strength in aged mice.

We also provide evidence that STEM administration robustly resulted in a higher total number of Pax7 + cells in aged muscle across all treatment groups when compared to PBS injected controls. Satellite cells are critical for regeneration following damage and are often dysfunctional in aged skeletal muscle; however, their role in skeletal muscle mass maintenance is debated [71–73]. A recent study highlighted the aging immune system as a modulator of the muscle stem cell environment [24]. For example, transplantation of old bone marrow cells into young mice decreased skeletal muscle Pax7 + cells and biased them toward a more fibrogenic cellular lineage [24]. Therefore, we suggest that STEM may modulate skeletal muscle immune cells thereby improve the aged muscle microenvironment and satellite cell function.

Lastly, the transcriptome analysis in C2C12 myotubes treated with STEM also support profound changes in gene networks related to skeletal muscle remodeling and collagen synthesis. However, the C2C12 analysis did not uncover any noteworthy changes to pathways governing immune cells as observed in the animal experiments. This is likely due to C2C12 myotubes not possessing any resident immune cells like the heterogenous cell nature of whole skeletal muscle [74]. This suggests that STEM, in addition to direct effects on muscle cells, may mediate immune cell content independent from direct targeted effects on the myofiber.

In summary, our results suggest that the novel secretome, STEM, improved aged skeletal muscle mass and strength and during disuse atrophy and recovery. While an exact mechanism of action is difficult to determine, we surmise that STEM’s unique growth factor properties and cytokine profile modulated immune and satellite cells that could possibly influence collagen turnover. We also recognize that STEM had isolated benefits in soleus compared to the other hindlimb muscle groups. Though STEM may preferentially target soleus muscle due oxidative muscle having heightened sensitivity to insulin and growth factors [75–77] and high macrophage content [78], we did notice some modest improvements in plantaris size in some experimental conditons with STEM treatment. This suggests that further optimization of dosing and delivery approaches will be important to advance STEM as a future therapeutic in aging muscle.

## Supplementary Information

Below is the link to the electronic supplementary material.

Top Expressed Factors in STEM Identified by Multiplex ELISA: Top identified immune modulators (red), growth factors (green) and cyto-skeletal factors (blue) in STEM. pg/ml denotes picograms per milliliter. All values are mean with standard error of the mean. Values were obtained via multiplex ELISA from 3 separate samples. (PDF 357 KB)

Raw Muscle Weight and Body Weight: Raw soleus (mg), plantaris (mg), gastrocnemius (mg), and bodyweight (g), for: (panel A, D) 14d ambulatory control PBS and STEM-treated mice, (panel B, E) 14d of HU PBS and STEM treated-mice, and (panel C, F) 7 day of recovery following 14d hindlimb unloading in PBS and STEM-treated mice. N=5 mice in each group. Results are mean with standard error of the mean. T-test between PBS and STEM. *=different to PBS. (PDF 59 KB)
