# Supplementary figures and images for: Reversal of deficits in aged skeletal muscle during disuse and recovery in response to treatment with a secrotome product derived from partially differentiated human pluripotent stem cells
Source: GeroScience. 2021 Aug 24;43(6):2635–52. doi: 10.1007/s11357-021-00423-0 (PMC8602548; doi:10.1007/s11357-021-00423-0)

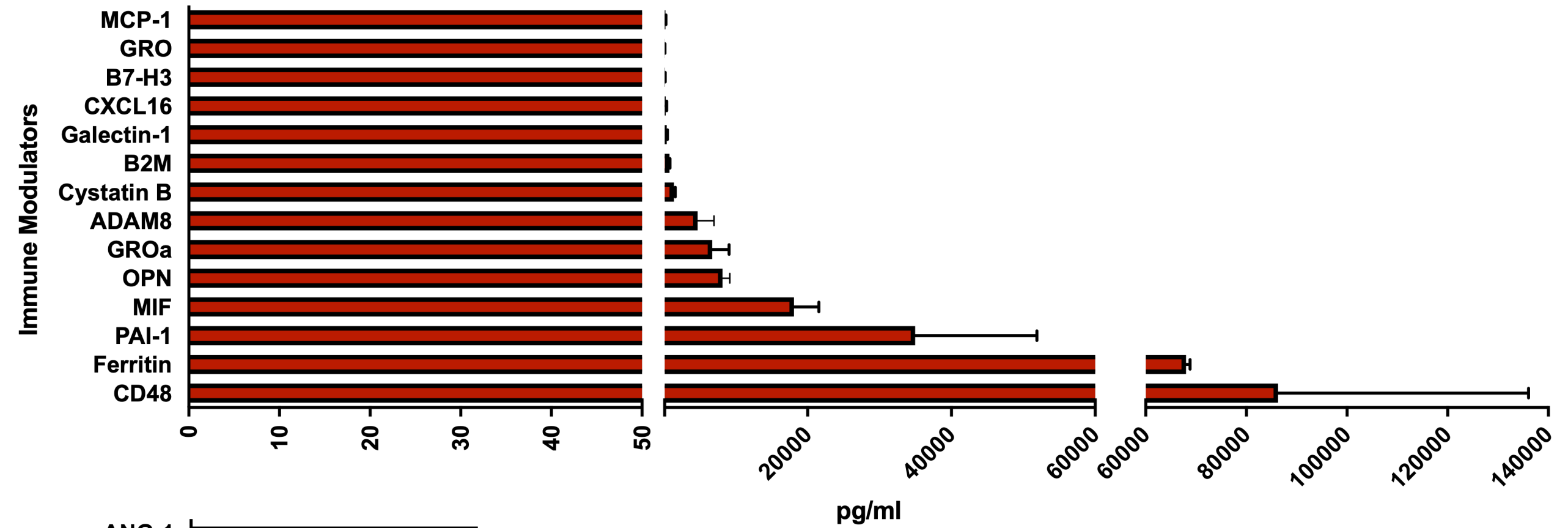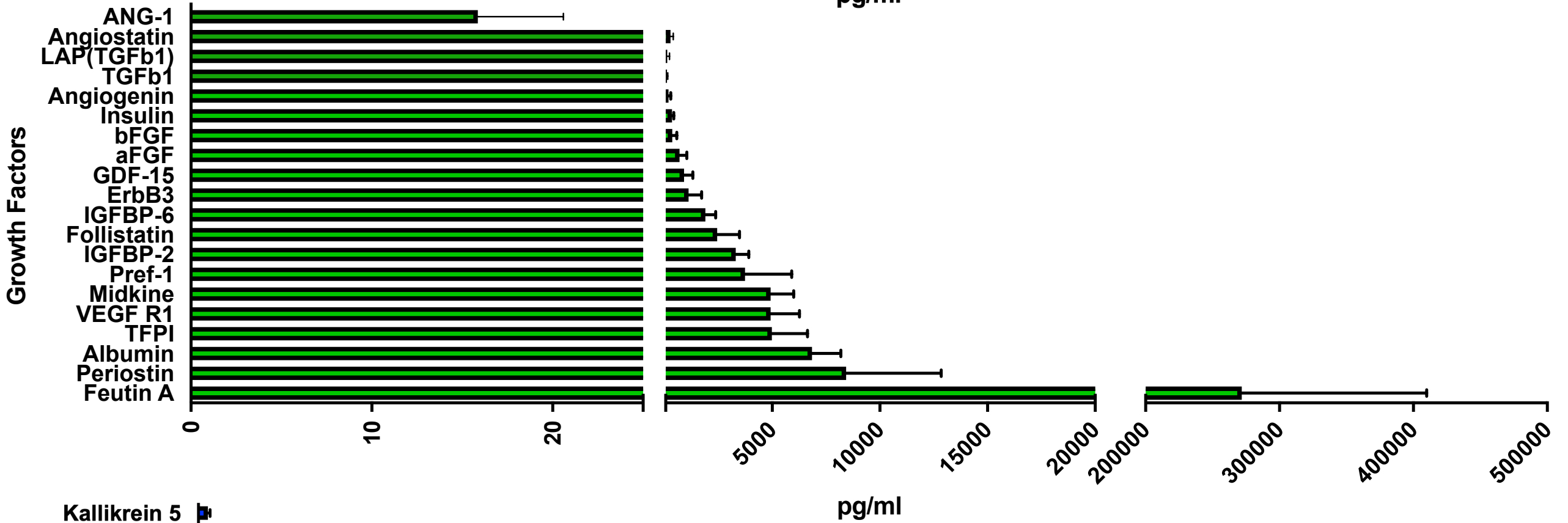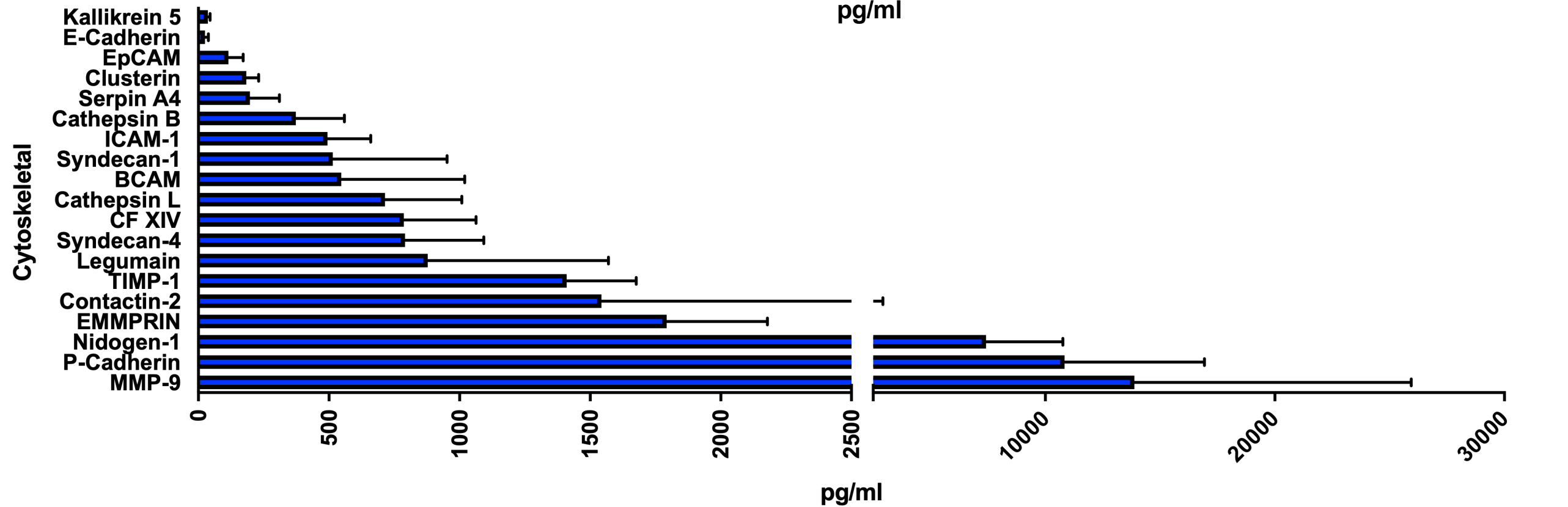

Supplement: Supplementary file 1 — Top Expressed Factors in STEM Identified by Multiplex ELISA: Top identified immune modulators (red), growth factors (green) and cyto-skeletal factors (blue) in STEM. pg/ml denotes picograms per milliliter. All values are mean with standard error of the mean. Values were obtained via multiplex ELISA from 3 separate samples. (PDF 357 KB) [file 11357_2021_423_MOESM1_ESM.pdf]

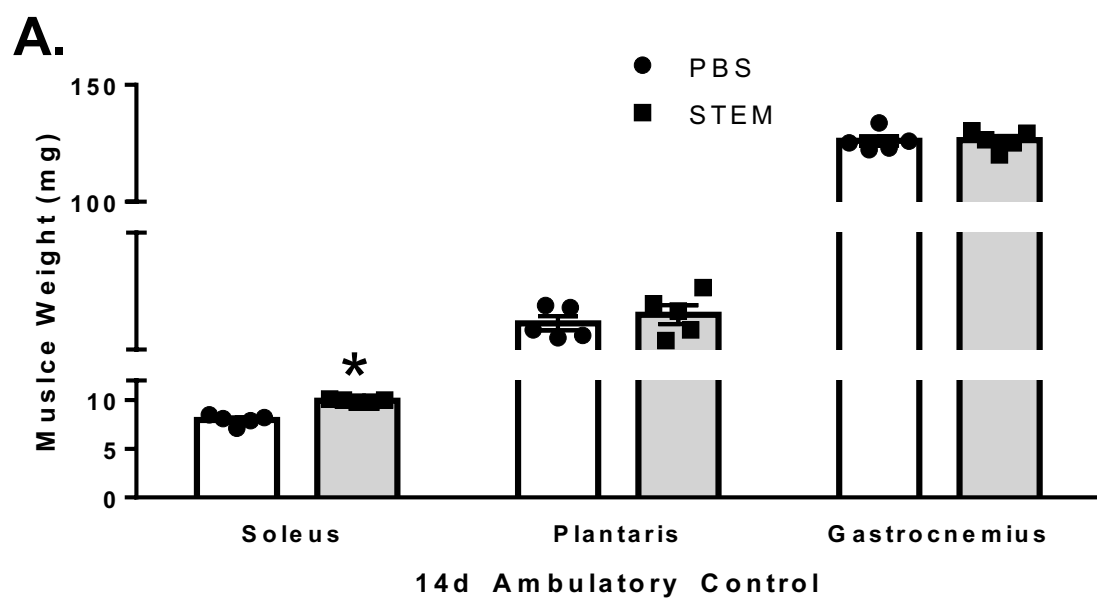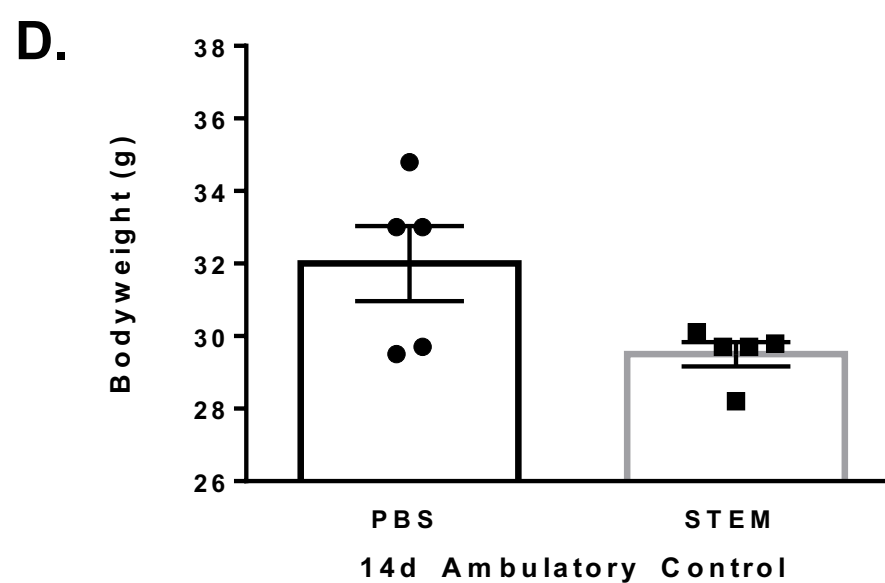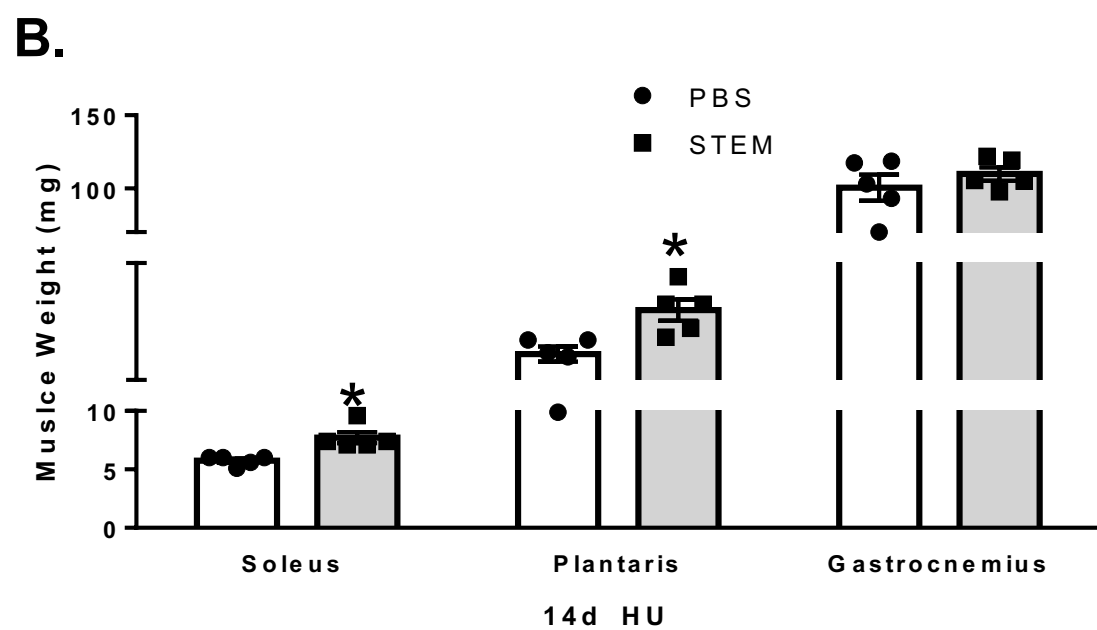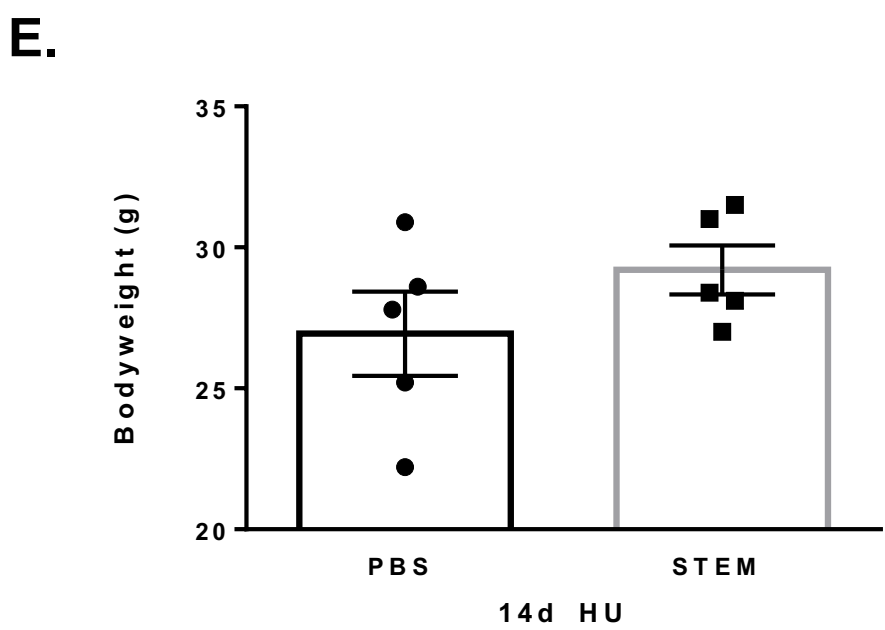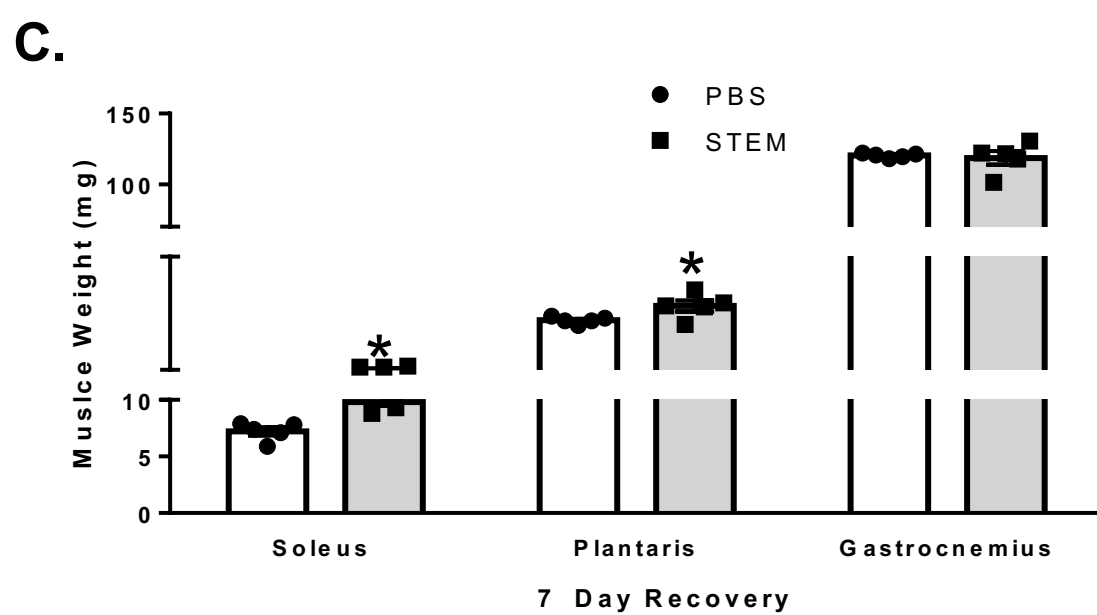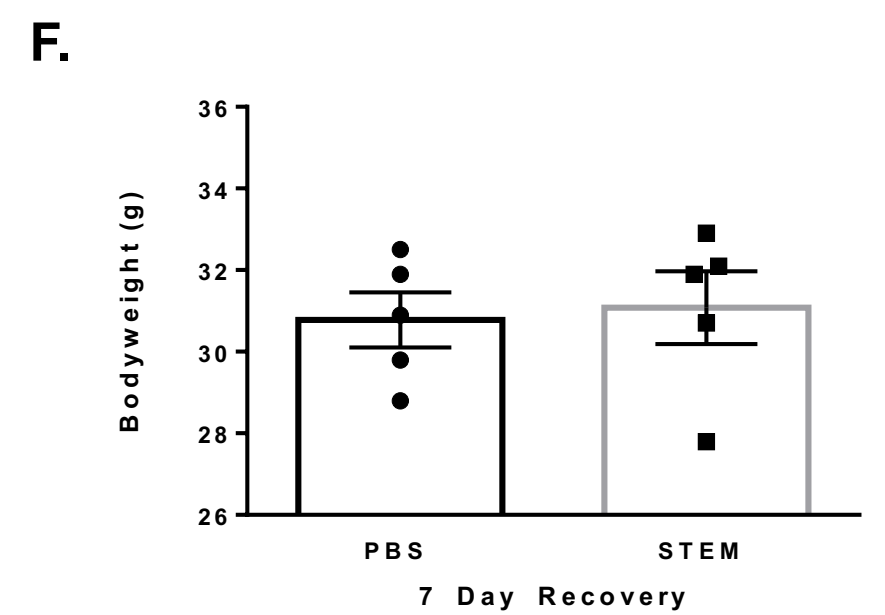

Supplement: Supplementary file 2 — Raw Muscle Weight and Body Weight: Raw soleus (mg), plantaris (mg), gastrocnemius (mg), and bodyweight (g), for: (panel A, D) 14d ambulatory control PBS and STEM-treated mice, (panel B, E) 14d of HU PBS and STEM treated-mice, and (panel C, F) 7 day of recovery following 14d hindlimb unloading in PBS and STEM-treated mice. N=5 mice in each group. Results are mean with standard error of the mean. T-test between PBS and STEM. *=different to PBS. (PDF 59 KB) [file 11357_2021_423_MOESM2_ESM.pdf]
